# Awareness and Knowledge of Parents and Caregivers Regarding Pediatric Obstructive Sleep Apnea in Jazan, Saudi Arabia: A Cross-Sectional Study

**DOI:** 10.7759/cureus.64297

**Published:** 2024-07-11

**Authors:** Ali Al-Makramani, Ali M Shawish, Ayman M Albarrati, Mohammed M Akkam, Nouf M Hakami, Rawan N Omar, Nouf M Oraibi, Atyaf M Mubaraky, Anisah M Humedi, Fuad A Sufyani, Alfdl A Khodh, Mohamed A Arishi, Nawal I Bahkali, Ibrahim A Mannaa, Anas E Ahmed

**Affiliations:** 1 Department of Pediatrics, Faculty of Medicine, Jazan University, Jazan, SAU; 2 College of Medicine, Jazan University, Jazan, SAU; 3 General Practice, Faculty of Medicine, Maastricht University, Maastricht, NLD; 4 Faculty of Medicine, Warsaw Medical University, Warsaw, POL; 5 General Practice, King Fahad Central Hospital, Jazan, SAU; 6 General Practice, Jazan University, Jazan, SAU; 7 General Practice, Jazan General Hospital, Jazan, SAU; 8 Community Medicine, Jazan University, Jazan, SAU

**Keywords:** jazan, saudi arabia, awareness, parental knowledge, pediatric obstructive sleep apnea

## Abstract

Background

Pediatric obstructive sleep apnea (POSA) is a common sleep disorder that can cause major health problems if untreated. Parental knowledge and awareness are critical for early detection and management of POSA in children. This study sought to measure parents' and caregivers' knowledge and awareness of pediatric POSA in Jazan, Saudi Arabia, as well as to uncover sociodemographic correlates of knowledge levels.

Methods

This cross-sectional study was conducted in Jazan, Saudi Arabia. A structured survey was used to collect data on sociodemographic factors and knowledge of pediatric OSA. Data were examined using descriptive statistics, ANOVA, and multivariate logistic regression analysis.

Results

The study included 393 parents and caregivers, 36.4% of whom were in the 18-30 year age group. Most were Saudis (n=385; 98%), and 212 (53.9%) were men. Most parents (n=378; 96.2%) had limited knowledge of pediatric OSA. Significant knowledge gaps were observed in recognizing POSA symptoms, understanding its impact on children's academic performance, and their association with mental health issues. Internet and social networking sites were the most common sources of knowledge about POSA (n=131; 33.33%), followed by knowing someone with OSA (n=103; 26.21%). Parents with more than eight children had a significant association with more knowledge than those without children (adjusted OR = 10.661, 95% CI: 1.367-83.139, p = 0.024).

Conclusion

The study identified significant gaps in the recognition of POSA symptoms and understanding its impact on their children's academic performance and mental health. Despite their limited knowledge, parents were receptive to early intervention for POSA. The study suggests that parents with more children had better knowledge of POSA, indicating that experiential learning may contribute to their understanding. The study recommends developing and assessing targeted education and awareness programs for parents, healthcare providers, and the public to improve POSA diagnosis, care, and outcomes.

## Introduction

Pediatric obstructive sleep apnea (POSA) is a sleep-associated disease defined by frequent partial or total obstructions of the upper airway during sleep. This results in disturbances of normal sleep patterns and breathing, resulting in sleep fragmentation and intermittent episodes of hypoxia and hypercapnia [1,2]. If left untreated, OSA can significantly impact quality of life, leading to several complications [2]. Symptoms and signs of the disease include daytime sleepiness, loud snoring, witnessed periods of breathing pauses during sleep, and exhaustion [3].

Obstructive sleep apnea (OSA) is the most common type of sleep apnea with a prevalence of 5% in the pediatric age group, most often due to adenoidal hypertrophy [4-6]. Although OSA is treatable, ignoring this condition can have a serious impact on the quality of life, due to resulting metabolic, neuro-cognitive, and cardiovascular disorders [7]. According to a study of early school-aged children, the risk of complication is associated with the severity of POSA, resulting in diminished cognitive and behavioral capacities affecting the development of optimal health. Patients with moderate to severe OSA and their relatives experience negative effects in their social and academic lives, affecting the entire community [8]. In another study, the people of the Asir region of Saudi Arabia were found to have little awareness of all elements of OSA, with most participants having no sources of information regarding the condition [9]. Similar findings were found in another study conducted in Saudi Arabia among primary care doctors, who were unaware of sleep medicine and the significance and effects of sleep disorders [10]. These results demonstrate the critical need for increasing awareness and enhancing education about this condition to both the public and medical professionals in Saudi Arabia.

This study's objective is to assess awareness and knowledge of OSA among parents and caregivers in Jazan and investigate potential factors associated with their knowledge level.

## Materials and methods

Study design and participants

We conducted a cross-sectional study to determine knowledge and awareness among parents regarding pediatric OSA from March 2024 to May 2024 in the Jazan region. This study included 393 respondents who participated in the survey. All Jazan region parents and caregivers were eligible for the study, only those who were able to answer were recruited. In this study, we included all parents and caregivers living in Jazan who are 18 years or older, whether they had children or not. We excluded all responses outside Jazan.

Sample size and data collection

The sample size for this study was calculated using the Raosoft sample size calculator (Raosoft Inc., Seattle, Inc.) [11], and 377 participants were needed to reach a 95% confidence interval and a 5% margin of error. After getting the consent form, the participant’s data were collected through an online survey distributed via social platforms to parents of Jazan, Saudi Arabia. Using the snowball convenient sampling technique, social media platforms were employed to reach our target sample, and parents were encouraged to distribute the survey.

Questionnaire

We used a validated and published questionnaire after obtaining their permission [7]. The questionnaire is composed of two parts. The first part includes sociodemographic information about our participants, including age, nationality, gender, marital status, education, occupation, and number of children. The second part contained questions assessing the knowledge regarding the definition, symptoms, risk factors, and treatment of OSA, also questions about the information source and the recommended method of raising awareness, and whether the respondents had children affected with OSA. Each question in the second part was given a weight based on its difficulty or importance, and the total knowledge score was calculated by adding up the weights for all correct answers. The maximum score was 30. Participants were divided into two groups based on their knowledge level: good knowledge (>70%) and poor knowledge (≤70%).

Data processing and analysis

Statistical analyses were conducted using Google Sheets and IBM SPSS Statistics v. 29 (IBM Corp., Armonk, NY). Descriptive statistics were computed to summarize the participants' sociodemographic characteristics and knowledge levels. These statistics comprised frequencies and percentages. The average knowledge scores of different sociodemographic categories were compared using one-way analysis of variance (ANOVA), as appropriate.

The study utilized multivariate logistic regression analysis to investigate the correlation between sociodemographic predictors and knowledge levels about pediatric OSA, with appropriate adjustments made to account for potential confounding variables. Initially, the model incorporated all sociodemographic variables; however, in the final model, the number of children was the sole predictor deemed significant. To quantify the strength of the association between knowledge levels and the number of children, adjusted odds ratios (aORs) and their respective 95% confidence intervals (CIs) were computed.

The comprehensive level of knowledge was classified as "good" or "poor" according to the cumulative knowledge score, with the researchers establishing the cut-off point. The prevalence of inadequate and adequate knowledge levels was illustrated as a percentage. In addition, descriptive statistics were employed to illustrate the frequencies and percentages of accurate responses pertaining to symptoms, risk factors, and suitable strategies for promoting awareness of pediatric OSA. Bar charts and tables were used to visually depict the results.

## Results

Table 1 presents the sociodemographic characteristics of the parents and their mean knowledge scores regarding pediatric OSA. The largest age group was 18-30 years (n=143; 36.4%). The vast majority (n=385; 98%) of the participants were Saudi nationals. Approximately half (n=212; 53.9%) of the participants were male, and most (n=302; 76.8%) were married. Over half (n=213; 54.2%) had a bachelor's degree.

**Table 1:** Sociodemographic characteristics and mean knowledge scores by sociodemographic variables. Statistically significant at the 0.05 level

Sociodemographic characteristics		N (%)	Knowledge score (Mean ± SD)	p-value	Test value
Age (years)	18-30	143 (36.4)	12.0 ± 6.1	0.853	F=0.337
	31-40	137 (34.9)	12.2 ± 4.5		
	41-50	85 (21.6)	12.7 ± 4.6		
	51-60	25 (6.4)	12.8 ± 3.2		
	>60	3 (0.8)	11.2 ± 4.0		
Nationality	Saudi	385 (98.0)	12.3 ± 5.1	0.413	T=0.820
	Non-Saudi	8 (2.0)	10.8 ± 6.7		
Gender	Male	212 (53.9)	11.7 ± 5.6	0.018*	T=-2.38
	Female	181 (46.1)	12.9 ± 4.4		
Marital status	Married	302 (76.8)	12.7 ± 5.0	0.006*	F=5.178
	Divorced	36 (9.2)	11.9 ± 4.7		
	Widowed	55 (14.0)	10.3 ± 5.6		
Education	High School	112 (28.5)	11.9 ± 5.1	0.006*	F=4.269
	Undergraduate	50 (12.7)	10.5 ± 5.2		
	Higher education	18 (4.6)	14.8 ± 3.7		
	Bachelor's degree	213 (54.2)	12.7 ± 5.1		
Occupation	Health sector employee	64 (16.3)	13.1 ± 5.3	0.275	F=1.259
	Engineer	37 (9.4)	11.4 ± 4.0		
	Teacher	81 (20.6)	12.8 ± 4.3		
	Soldier	51 (13.0)	11.0 ± 5.4		
	Pilot	1 (0.3)	16.5 ± 0		
	Lawyer	4 (1.0)	11.6 ± 3.5		
	Other	155 (39.4)	12.3 ± 5.5		
No. of children	No children	126 (32.1)	11.6 ± 5.7	0.021*	F=3.283
	1-3	171 (43.5)	12.1 ± 4.6		
	4-7	84 (21.4)	13.2 ± 4.4		
	8 or more	12 (3.1)	15.4 ± 7.4		
Total knowledge score; Mean ± SD		12.3 ± 5.1			

Females had higher mean knowledge (12.9 ± 4.4) than males (11.7 ± 5.6) [p = 0.018]. Married parents had higher knowledge (12.7 ± 5.0) than divorced (11.9 ± 4.7) or widowed (10.3 ± 5.6) parents [p = 0.006]. Parents with higher education degrees had higher knowledge scores, with a mean of 14.8 ± 3.7 compared to 10.5 ± 5.2 for undergraduates [p = 0.006]. Parents with eight or more children had the highest mean knowledge score (15.4 ± 7.4) compared to those with no children (11.6 ± 5.7) [p = 0.021]. The mean overall knowledge score was 12.3 ± 5.1 out of a maximum score of 30.

Only 175 (44.5%) of parents could correctly identify the definition of OSA in children as "episodes of recurrent sleep apnea." Of note, 127 (32.3%) reported that they did not know the definition. Regarding the prevalence of POSA among their own children, 118 (30%) of parents reported having a child with POSA and snoring. The data reveal a lack of knowledge about POSA among parents. An overwhelming majority (n=378; 96.2%) had poor knowledge levels based on their total knowledge scores, whereas only 15 (3.8%) demonstrated good knowledge (Table 2).

**Table 2:** Knowledge of POSA definition and prevalence of POSA POSA: pediatric obstructive sleep apnea

Statement		N	%	p-Value	Test value
What is obstructive sleep apnea in children?	A natural phenomenon that occurs during sleep	64	16.3%	P<0.001	X^2^=131.97^a^
	Complete interruption of breathing throughout the period of sleep until the time of waking up	27	6.9%		
	Episodes of recurrent sleep apnea	175	44.5%		
	I don't know	127	32.3%		
Do you have a boy or girl who suffers from sleep apnea and snoring?	No children	89	22.6%	P<0.001	X^2^=37.84^b^
	No	186	47.3%		
	Yes	118	30.0%		
Level of knowledge	Poor	378	96.2%	P<0.001	X^2^=335.29^a^
	Good	15	3.8%		

Approximately half of the parents (n=211; 53.7%) were unaware that OSA in children can affect their educational performance. A majority of 224 (57%) did not know that children with OSA had a higher risk of depression than the others. However, most parents (n=306; 77.9%) believed that POSA could be treated (Table 3).

**Table 3:** Percentages and frequencies of general knowledge answers

General knowledge		N (%)	p-Value	Test value
Did you know that obstructive sleep apnoea in children affected by it affects their educational performance?	No	211 (53.7)	P=0.144	X^2^=2.14^a^
	Yes	182 (46.3)		
Did you know that children with obstructive sleep apnoea have a higher risk of depression than others?	No	224 (57.0)	P=0.006	X^2^=7.69^a^
	Yes	169 (43.0)		
Did you know that obstructive sleep apnoea in children with it affects their attention and behaviour?	No	196 (49.9)	P=0.960	X^2^=.003^a^
	Yes	197 (50.1)		
Do you think that genes play a role in causing obstructive sleep apnoea in children?	No	190 (48.3)	P=0.512	X^2^= .430^a^
	Yes	203 (51.7)		
Do you think that obstructive sleep apnoea in children can be treated?	No	87 (22.1)	P<0.001	X^2^=122.03^a^
	Yes	306 (77.9)		
Do you think that early treatment and early diagnosis can reduce the incidence of potential complications in children with obstructive sleep apnoea?	No	69 (17.6)	P<0.001	X^2^=165.45^a^
	Yes	324 (82.4)		
Do you think that parents’ awareness of obstructive sleep apnoea in children helps reduce the burden on families and thus on society as a whole?	No	71 (18.1)	P<0.001	X^2^=165.45^a^
	Yes	322 (81.9)		

When we asked about the symptoms of OSA, the most commonly reported symptoms were snoring (n=210; 53.44%), discomfort during sleep (n=200; 50.89%), mouth breathing (n=183; 46.56%), and waking from sleep (n=181; 46.06%). Other notable symptoms included frequent coughing or choking (n=113; 28.75%), noticeable apnea episodes (n=111; 28.24%), excessive daytime sleepiness (n=46; 11.70%), nightmares (n=44; 11.20%), hyperactivity (n=32; 8.14%), and nocturia (n=29; 7.38%).

**Figure 1:**
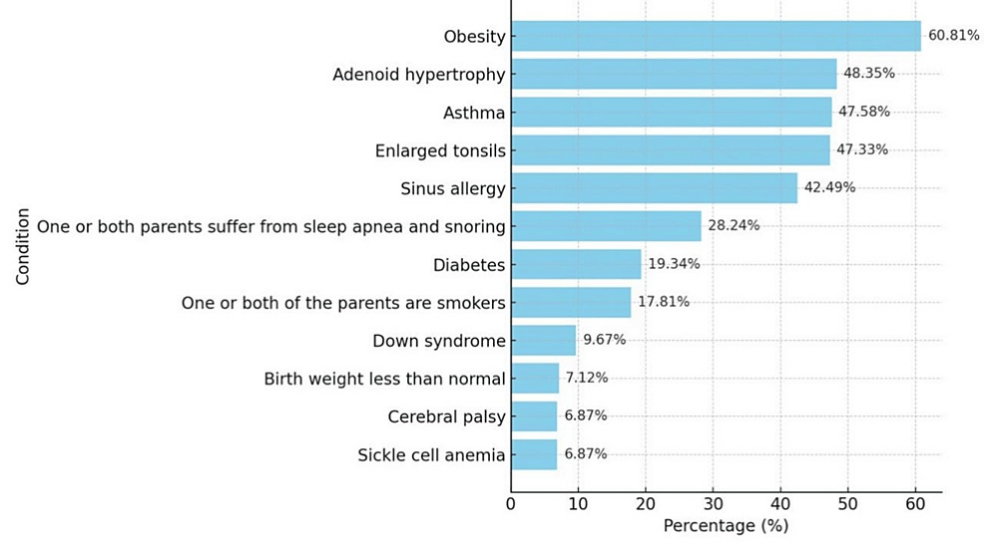
The percentages of correct answers regarding the risk factors of POSA POSA: pediatric obstructive sleep apnea

When it came to risk factors for POSA as shown in Figure 1, the majority of parents correctly identified obesity (n=239; 60.81%), adenoid hypertrophy (n=190; 48.55%), asthma (n=187; 47.58%), enlarged tonsils (n=186; 47.33%), and sinus allergies (n=167; 42.49%) as risk factors. 

Table 4 presents the results of the multivariate logistic regression analysis examining the predictors of knowledge about POSA among parents. All the sociodemographic factors were entered into the model. In univariate analysis (ANOVA), the number of children emerged as a statistically significant predictor of parents' knowledge levels regarding POSA. This variable remained significant in the subsequent multivariate regression model, after adjusting for other sociodemographic variables. Specifically, parents with eight or more children had significantly higher odds of having good knowledge about POSA than parents without children (adjusted OR = 10.661, 95% CI: 1.367-83.139, p = 0.024).

**Table 4:** Predictors of knowledge about POSA Multivariate logistic regression analysis

Variable*	P-Value	aOR	95% C.I.for aOR	
			Lower	Upper
Number of children, Reference = No children				
1-3 children	0.983	0.984	0.210	4.604
4-7 children	0.305	2.712	0.404	18.221
8 or more children	0.024	10.661	1.367	83.139

When we asked about the sources of knowledge about POSA, a significant proportion of 150 (38.17%) of parents reported not having heard of it. The most common sources of information were the Internet and social networking sites (n=131; 33.33%), followed by knowing someone affected by OSA (n=103; 26.21%) and medical articles (n=87; 22.14%).

When we asked about the most appropriate ways to raise awareness regarding OSA in children, consulting a specialist doctor was the top recommendation, endorsed by 316 (80.41%) of the respondents. More than half (n=215; 54.71%) believed that voluntary awareness campaigns could be effective in increasing knowledge. Additionally, 177 (45.04%) viewed the Internet and social networking sites as useful platforms for disseminating information about POSA.

## Discussion

This study found considerable information gaps among parents, with an alarming 96.2% having little overall understanding of POSA. These results are consistent with those of other studies that have indicated low levels of parental knowledge and awareness of POSA [12,13]. Insufficient understanding can result in delayed identification and treatment of POSA, potentially causing long-term effects on children's health, cognitive development, and quality of life [14]. The study revealed that parents had insufficient awareness regarding the symptoms of POSA, comprehending its effects on children's academic performance and its correlation with mental health problems, including depression. These knowledge gaps are worrisome because they can prevent parents from promptly receiving medical care for their children. POSA symptoms such as snoring, gasping, and daytime sleepiness are frequently ignored or confused with typical childhood behaviors [15]. However, these symptoms may suggest severe underlying sleep issues that necessitate immediate treatment. Parents may not immediately recognize the effects of POSA on children's academic performance and mental health, causing a delay in treating these issues [16]. These results emphasize the necessity of specific educational measures to enhance parental understanding of POSA symptoms and their possible effects on children's health and well-being [15,16].

Despite their limited understanding, parents showed a good attitude toward the significance of early intervention and management of OSA in children. They also recognized the importance of parental awareness in alleviating the impact of POSA on families and society. These results indicate that parents are open to educational initiatives and can be included as proactive collaborators to oversee their children's sleep well-being. Prior research has highlighted the significance of parental participation in effectively managing POSA [17,18]. When parents have a good understanding of POSA and its treatment choices, they are more inclined to follow suggested interventions such as lifestyle changes, drugs, or surgeries. Parental engagement helps guarantee that children receive essential follow-up care and monitoring to prevent the reappearance of POSA symptoms [18]. The survey found that parents most obtained information on POSA from the Internet and social networking platforms. This discovery emphasizes the increasing significance of digital health information and the possibility of using online platforms to distribute precise and evidence-based information regarding POSA [19]. It is essential to guarantee that parents have access to reliable and trustworthy sources of internet health information because of the significant variability in quality and reliability [20]. Healthcare providers and public health groups should actively participate in creating and endorsing evidence-based online tools that offer precise and easily available information about POSA. They should interact with parents on social media to correct misunderstandings and offer advice on when to seek medical help for their children's sleep problems [19].

Multivariate logistic regression analysis showed that the number of children was the sole significant sociodemographic predictor of knowledge level. Parents with eight or more children exhibited notably superior awareness of POSA compared to parents with no children. Experiential learning and exposure to child health issues in larger families may lead to greater knowledge and awareness of POSA. Prior research has found comparable connections between parental expertise and understanding of child health issues [21,22]. Parents with several children may have had more opportunities to notice and deal with sleep problems in their children, resulting in a deeper comprehension of POSA and its symptoms. Parents with larger families may have increased interactions with healthcare practitioners and access to more information about child health issues such as sleep disorders [21].

The findings of this study have significant implications for clinical practice and public health. Healthcare professionals should prioritize POSA during well-child visits and provide parents with detailed information about the illness. This involves informing parents about the signs and symptoms of POSA, its possible effects on children's health and growth, and treatment choices [7]. Healthcare practitioners should assess children for risk factors of POSA, including obesity, craniofacial anomalies, and neuromuscular diseases, and recommend additional examination and treatment if necessary [14]. Public health efforts are necessary to increase awareness of pediatric OSA and encourage healthy sleep habits in children and families along with therapeutic measures. Customized educational materials, including brochures, posters, and online resources, should be created, and distributed to tackle the prevalent misunderstandings and lack of information regarding POSA [7]. Information should be culturally sensitive and easily understood by parents with different levels of health literacy. Public health campaigns can be used to emphasize the significance of sleep health and encourage parents to seek medical help for their children's sleep problems [23].

This study highlights the opportunity to use digital platforms such as social media and the Internet to reach a broader audience and enhance parental understanding and awareness of POSA. Utilizing evidence-based internet tools and social media campaigns can effectively distribute correct information and involve parents in managing their children's sleep health [23,24]. Healthcare providers and public health organizations should work together with digital health specialists to create and execute these plans, ensuring that they are grounded in current scientific research and effective health communication methods [19].

Future studies should focus on creating and assessing the impact of educational programs and awareness initiatives aimed at parents and caregivers, healthcare professionals, and the public. Longitudinal studies offer useful insights into how enhancements in parental knowledge and awareness lead to improved POSA diagnosis, management, and outcomes in children [25,26]. These studies should investigate the enduring effects of POSA on children's health, cognitive development, and quality of life, along with an analysis of the cost-effectiveness of various intervention techniques.

There are limitations to this study that need to be considered when analyzing the results. The cross-sectional design did not allow for causal inferences, and the sample was restricted to parents in Jazan, Saudi Arabia, thereby restricting the applicability of the results to other populations. The study depended on self-reported data, which could be influenced by recall and social desirability biases.

## Conclusions

This study was conducted among parents of the Jazan region. The study aimed to assess parents' awareness and knowledge about POSA. The study identified significant gaps in recognizing POSA symptoms and understanding their impact on children's academic performance and mental health. Despite the limited knowledge, parents were receptive to early intervention for POSA. The study suggests that parents with more children had better knowledge of POSA, indicating that experiential learning may contribute to their understanding. The study recommends developing and assessing targeted education and awareness programs for parents, healthcare providers, and the public to improve POSA diagnosis, care, and outcomes.

## Appendices

The questionnaire we used was developed by Alosaimi et al., and their permission was obtained to use the validated version in this study [7] (Table 5).

**Table 5:** Questionnaire

Age	18-30
	31-40
	41-50
	51-60
	More Than 60
Nationality	Saudi
	Non-Saudi
Sex	Male
	Female
Marital Status	Married
	Widowed
	Divorced
Educational Level	High School
	Undergraduate
	Higher Education
	Bachelor's Degree
Occupation	Health Sector Employee
	Engineer
	Teacher
	Soldier
	Pilot
	Lawyer
	Other
Number Of Children	No Children
	1-3
	4-7
	8 Or More
What Is Obstructive Sleep Apnea In Children? (One Answer Only)	Episodes Of Recurrent Sleep Apnea*
	Complete Interruption Of Breathing Throughout The Period Of Sleep Until The Time Of Waking Up
	A Natural Phenomenon That Occurs During Sleep
	I Don’t Know
Do You Have A Boy Or Girl Who Suffers From Sleep Apnea And Snoring?	No Children
	Yes
	No
How Did You Know About The Problem Of Obstructive Sleep Apnea In Children? (You Can Choose More Than One Answer)	I Had Not Heard Of It
	The Internet And Social Networking Sites
	A Person Who Already Suffers From Sleep Apnea
	Medical Articles
	Other
If A Child Suffers From Obstructive Sleep Apnea, What Are His Symptoms Expected To Be? (You Can Choose More Than One Answer)	Snoring
	Discomfort In Sleep
	Mouth Breathing
	Waking Up From Sleep
	Frequent Coughing And Choking
	Noticeable Episodes Of Apnea During Sleep
	Excessive Daytime Sleepiness
	Nightmares
	Hyperactivity
	Nocturia
Did You Know That Obstructive Sleep Apnoea In Children Affected By It Affects Their Educational Performance?	Yes
	No
Did You Know That Children With Obstructive Sleep Apnoea Have A Higher Risk Of Depression Than Others?	Yes
	No
Did You Know That Obstructive Sleep Apnea In Children With It Affects Their Attention And Behavior?	Yes
	No
Do You Think That Genes Play A Role In Causing Obstructive Sleep Apnoea In Children?	Yes
	No
Do You Think That Obstructive Sleep Apnoea In Children Can Be Treated?	Yes
	No
Do You Think That Early Treatment And Early Diagnosis Can Reduce The Incidence Of Potential Complications In Children With Obstructive Sleep Apnoea?	Yes
	No
Do You Think That Parents’ Awareness Of Obstructive Sleep Apnoea In Children Helps Reduce The Burden On Families And Thus On Society As A Whole?	Yes
	No
What Do You Think Are The Risk Factors For Sleep Apnea And Snoring In Children? (You Can Choose More Than One Answer)	Obesity
	Adenoid Hypertrophy
	Asthma
	Enlarged Tonsils
	Sinus Allergy
	One Or Both Parents Suffer From Sleep Apnea And Snoring
	Diabetes
	One Or Both Of The Parents Are Smokers
	Down Syndrome
	Birth Weight Less Than Normal
	Cerebral Palsy
	Sickle Cell Anemia
In Your Opinion, What Is The Most Appropriate Way To Raise Awareness In Society About Obstructive Sleep Apnea In Children? (You Can Choose More Than One Answer)	Consult A Specialist Doctor
	Voluntary Awareness Campaigns
	The Internet And Social Networking Sites
